# Conservation, Divergence, and Genome-Wide Distribution of *PAL* and *POX A* Gene Families in Plants

**DOI:** 10.1155/2013/678969

**Published:** 2013-03-10

**Authors:** H. C. Rawal, N. K. Singh, T. R. Sharma

**Affiliations:** Genoinformatics Laboratory, National Research Centre on Plant Biotechnology, Indian Agricultural Research Institute, Pusa Campus, New Delhi 110 012, India

## Abstract

Genome-wide identification and phylogenetic and syntenic comparison were performed for the genes responsible for phenylalanine ammonia lyase (PAL) and peroxidase A (POX A) enzymes in nine plant species representing very diverse groups like legumes (*Glycine max* and *Medicago truncatula*), fruits (*Vitis vinifera*), cereals (*Sorghum bicolor*, *Zea mays*, and *Oryza sativa*), trees (*Populus trichocarpa*), and model dicot (*Arabidopsis thaliana*) and monocot (*Brachypodium distachyon*) species. A total of 87 and 1045 genes in PAL and POX A gene families, respectively, have been identified in these species. The phylogenetic and syntenic comparison along with motif distributions shows a high degree of conservation of PAL genes, suggesting that these genes may predate monocot/eudicot divergence. The POX A family genes, present in clusters at the subtelomeric regions of chromosomes, might be evolving and expanding with higher rate than the PAL gene family. Our analysis showed that during the expansion of POX A gene family, many groups and subgroups have evolved, resulting in a high level of functional divergence among monocots and dicots. These results will act as a first step toward the understanding of monocot/eudicot evolution and functional characterization of these gene families in the future.

## 1. Introduction

All plant species are continuously fighting with different biotic and abiotic stresses for their existence. The stresses like harsh environmental conditions, desiccation, UV radiation, and attack of microbial pathogens may affect growth and development in the plants and sometimes lead to their death. Although all plants have different natural defense mechanisms against these stresses, in most cases, plants activate the phenylpropanoid pathway in response to pathogen attack or to elicitors [1]. In the plant phenylpropanoid pathway, phenylalanine ammonia lyase (PAL) is a key enzyme that catalyses the first step in the pathway and not only leads to the accumulation of phytoalexins [2] but also contributes in growth and development of plants and responses to biotic stresses [3, 4].

The plant peroxidase (POX) genes are heme-containing glycoproteins present in large numbers in higher plants [5]. These genes are involved in defense against pathogen infection or insect attack, and several other physiological functions such as H_2_O_2_ removal, toxic reduction, oxidation, lignification, suberization, auxin catabolism, and wound healing in plants [5, 6]. Plants contain multiple isoforms for peroxidases, which respond to stresses in different or similar ways by making POX genes an important one for self-defense in plant tissues against various biotic stresses including pathogen infection [7]. The plant peroxidase super family can be further divided into three classes (classes I, II, and III) based on their structural differences [8]. Among these, class III plant peroxidases (EC 1.11.1.7) were originally described as peroxidases. Two wound-inducible peroxidase genes, that is, POX A and POXN have been reported, and only POX A has been functionally validated in transgenic rice plants showed xylem-specific expression and was conserved between dicot and monocot species [9]. Hence, the present study was focused on the peroxidase gene, *POX A* for identification and exploring its syntenic relationship in dicots and monocots.

Because phenylpropanoid pathway genes and defense-response genes are highly regulated in the infection process [10], these need to be studied in detail at the whole genome level. Little is known about their organization and evolution in the plants. Earlier, many researchers have contributed by comparative analysis of one or the other important gene families like DNA binding with one finger (Dof) [11], cystatin [12], pentatricopeptide repeat (PPR) [13], squamosa promoter-binding protein (SPB) [14], and prohibitin (PHB) [15]. However, these studies were restricted to genomic distribution and phylogenetic analysis of two or three species and hardly discussed about their syntenic relations.

The pattern of gene distribution of a specific gene family across the genomes is very important in understanding the evolutionary history of the genes. Telomeres, the natural ends of eukaryotic chromosomes, are critical in conserving genetic integrity [16]. Subtelomeres are the chromosome regions found immediately internal to the telomere regions, which are characterized by the presence of genes amplified through the spread to multiple chromosome ends, resulting in subtelomeric gene families [17, 18]. Large blocks of genes usually present in these subtelomeric ends, which dispersed to different chromosomes during crossing over, resulting in extensive sequence variation [19]. It has also been shown that the rate of evolution of these subtelomeric gene families is high compared with the genes present in other regions of the genome [20].

The availability of complete genome sequences of important plant species enables us to compare and analyze their evolution by correlating genes with respect to their relative positions in the genomes, understanding their phylogenetic relation, and detecting the percentage of orthology shared between the genomes. In many areas of bioinformatics such as comparative genomics, synteny analysis is currently an important part of data analysis. If we are looking to unravel the history of a gene family, the accurate relation between genes in the gene family must be determined across the species of interest. These relations can be described either in terms of orthology or paralogy, which are two key concepts of evolutionary genomics [21]. The orthologs are genes that diverged because of speciation event, whereas in paralog sequence, divergence follows gene duplication [22]. Hence, orthologs are the genes that, at present, exist in different species but earlier have originated from a single gene in the last common ancestor of these species and have often retained identical biological functions [23]. One can also conclude that the fundamental function of orthologous pairs/groups may have been conserved across evolutionary related species. The percentage of orthologous pairs between genomes of evolutionary related species can be used to infer their synteny relations. The high percentage of orthology for a gene family between two species may reflect high conservation of their function in those species [13]. This type of genome-wide analysis has not been performed across monocot and dicot species. The objectives of the present study were as follows: (i) to compare and analyze genome-wide evolution of PAL and POX A genes with respect to their relative positions in the plant genomes, (ii) to understand their phylogenetic relation, and (iii) to understand orthologous relation between different monocot and dicot genomes.

## 2. Materials and Methods

### 2.1. Genes and Genomes Used in This Study

The availability of the whole genome and abundant genetic and genomic resources of *Oryza sativa* (rice) with high-syntenic relationships with other plant genomes, makes it a better option for comparative genome analysis [24–26]. We have selected the cloned and characterized genes of phenylalanine ammonia lyase (PAL) (accession number X16099.1; 701 amino acids) and POX gene for peroxidise (POX A) (accession number D84400.1; 326 amino acids) from *Oryza sativa* as query for our genome-wide analysis and comparative studies of these two DR gene families. We have downloaded their amino acid sequence from the National Centre for Biotechnology Information (NCBI). We included the representative nine plant genomes (five dicots and four monocots) in this study for comparative analysis. These nine plant species also represent very diverse groups like legumes (*Glycine max* and *Medicago truncatula*), fruits (*Vitis vinifera*), cereals (*Sorghum bicolor*, *Zea mays*, and *Oryza sativa*), trees (*Populus trichocarpa*), and model dicot and monocot species (*Arabidopsis thaliana* and *Brachypodium distachyon*). All genes, ESTs, and whole genome sequences which were downloaded for each of these species from different resources are given in Table 1.

### 2.2. Identification of *PAL* and *POX A* Genes

For each plant species, PAL and POX A genes were identified by systematic BLAST [22] searches of each of the query gene sequence against the gene sequences of all nine plant species separately. For each and every BLAST search, BLAST default settings were used, and BLAST hits were considered significant with bit score ≥100 and E-value ≤*e* ^−20^. *In silico* expression analysis was conducted for these identified genes by BLAST search against the EST sequences of respective plant species downloaded in local database. For each gene, we counted the number of significant EST hits (those having bit score ≥100 and E-value ≤*e* ^−20^) and categorized these genes as “not expressed,” “less expressed,” “moderately expressed,” and “highly expressed” if there was no hit, 1 to 100 hits, 101 to 400 hits, and more than 400 hits, respectively.

### 2.3. *In Silico* Physical Mapping

The chromosomal position was detected for each of the PAL and POX A genes identified by BLAST search against full genome sequence of the respective plant species. BLAST results were parsed with in-house developed Perl scripts and with excel worksheets and formulae to detect the exact position on chromosomes for each of the identified gene. The physical maps were then prepared using Mapchart 2.2 [27] to map these positions of genes on the chromosomes. The position of each gene was represented in base pairs.

### 2.4. Phylogenetic Analysis

The neighbour-joining (NJ) method was preferred by the earlier researcher because of its reasonable accuracy and cubic running time which makes this method a widely used one for phylogenetic tree construction [28, 29]. The multiple sequence alignment (MSA) of all identified PAL and POX A genes was performed to construct a phylogenetic tree by ClustalX 2.1 using default parameters [30]. The neighbour-joining distance trees were constructed separately for both the gene families using default settings and 2000 bootstrap replications to ensure a high confidence range and accuracy [31]. Bootstrap analysis was performed to evaluate the degree of support for each homologous group in the tree.

The first tree prepared was that for 87 PAL sequences by ClustalX 2.1, and that was further supported by MEME 4.6.0 [32] results. Multiple Expectation-Maximization for Motif Elicitation (MEME) is a suite of tools for motif discovery and searching. This suite is quite often used by previous researchers for the support of phylogenetic trees and to find the conserved motif structures. About twenty different subdomains or motifs between 6 and 50 residues were detected and distributed by MEME software. An overlay of phylogenetic tree and motif distribution from MEME can be used to find the correlation [12, 15]. The trees are thus found to be correlated and well supported, then further represented interactively using iTOL (Interactive Tree OF Life), an online tool for the display and curation of phylogenetic trees [33]. Similarly, phylogenetic tree was also constructed for 1045 POX A genes by ClustalX 2.1 and represented interactively using iTOL.

### 2.5. Synteny Analysis

We used the orthology information to infer the synteny between each of the nine plant genomes for both PAL and POX A genes. The “best bidirectional hit” (BBH) method has been the most frequently applied method to determine orthologous pairs [34]. However, because of changing mutation rates over evolutionary time and the approximate nature of BLAST, the method based on just top BLAST hit can very often might miss some possible orthologous pairs. Also, these methods provide only one-to-one relations for the orthologous pairs. One of our objectives was to find out all possible orthologous pairs to find one correct ortholog [35, 36]. However, a single clear ortholog can be identified for each of these genes because of the numerous changes in the monocots and dicots; due to their divergence, most genes may have more than one “ortholog.” Hence we employed this method for getting one-to-many relations.

As InParanoid program (INP method) is the best ortholog identification method in terms of identifying functionally equivalent proteins [34] and provides one-to-many relations, we decided to use a combined approach of these two methods with higher threshold values. This combined approach of the BBH and INP methods was used to find out the number of possible orthologous pairs for PAL and POX A genes in each of the nine plant species included in this study.

To be defined as orthologs, gene pairs must meet several criteria including sequence identity and conservation of function at the level of expression and activity [37]. Because the PAL and POX A genes are involved in the defense mechanism of plants, their sequence identity becomes an important criteria. For determination of orthologs, we performed all-against-all BLAST search of the genes of one genome against the other. We used a three-scale parameter or threshold to filter out the significant hits. These parameters were the BLAST bit score ≥100, E-value ≤*e* ^−20^ and 20% identity between amino acid sequences over at least 50% of the protein length. Any two significant BLAST hits that match the afore-mentioned criteria and have bidirectional hits with each other were considered as orthologs to each other and were counted as single orthologous pair [38].

We performed parsing of the all-against-all BLAST search output, processing, and detection of the orthologous pairs by using some in-house developed Perl scripts and with excel worksheets and formulae. The percentage was then calculated by taking into account the total number of possible orthologous pairs (product sum of the number of PAL or POX A genes among the two plant genomes of interest) and the number of orthologous pairs found in the analysis. The percentage of orthologous pairs was tabulated and then represented in the form of figures by Circos software [39].

## 3. Results and Discussion

### 3.1. *PAL* and *POX A* Genes in Dicots and Monocots

We performed genome-wide analysis of the PAL and POX A genes in nine plant species (Table 1) that not only represent two major classes such as dicots and monocots but also belong to very diverse groups like legumes (*Glycine max* and *Medicago truncatula),* fruits (*Vitis vinifera*), cereals (*Sorghum bicolor*, *Zea mays*, and *Oryza sativa*), trees (*Populus trichocarpa*), and model dicot and monocot species (*Arabidopsis thaliana* and *Brachypodium distachyon*). This study revealed a well-known fact that, on average, monocots have larger genome size than dicots (Table 1). Because of their divergence, the different evolutionary paths of the monocots and dicots might have been important for the induction of variations in genome size and number of genes via genome shuffling [40, 41]. On average, the dicots used in this study were found to have a genome size of 391.76 Mb compared with that of 840.66 Mb average genome size of the monocots. Furthermore, it was observed that five dicots used in present study have a higher number of chromosomes compared with that of four monocots. Even in earlier reports, a negative correlation was observed between the genome size and basic chromosome number of the monocots and dicots [34].

**Figure 1:**
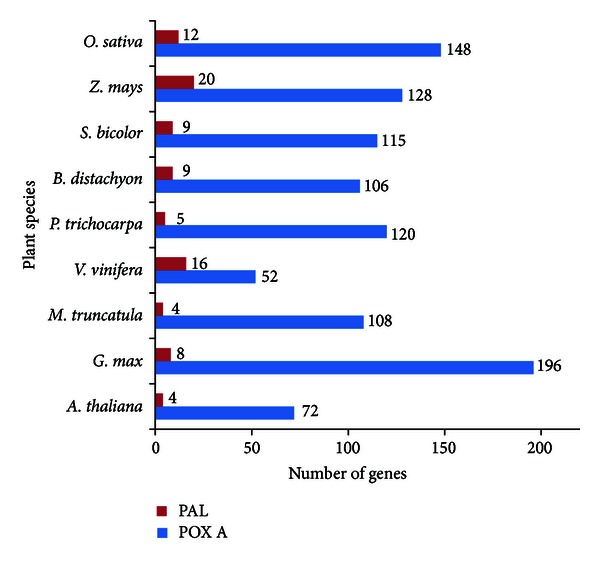
PAL and POX A genes identified in different plant species. The number at the top of the bar indicates the number of identified PAL (red) genes and *POX A* (blue) genes.

We identified a total of 87 PAL and 1045 POX A genes in the nine plant species included in this study regardless of their genome sizes (Figure 1). The maximum number (196) of POX A genes was found in case of *Glycine max*, whereas the minimum number (52) was found in *Vitis vinifera*. The monocots were found to have more number of PAL genes as compared with dicots. There were 12 PAL genes in rice and only four in *Arabidopsis thaliana*, which has also been reported earlier [4]. The number of POX A genes identified in this study for Arabidopsis (72), rice (148), and other plants species is quite comparable with the earlier reports [42, 43] despite the fact that different approaches have been used in both studies. With just 87 in number, there seems hardly any expansion in the PAL gene family after monocot/eudicot divergence. Although with 1045 identified genes, the POX A family seems to expand with higher rate than the PAL gene family.

**Figure 2:**
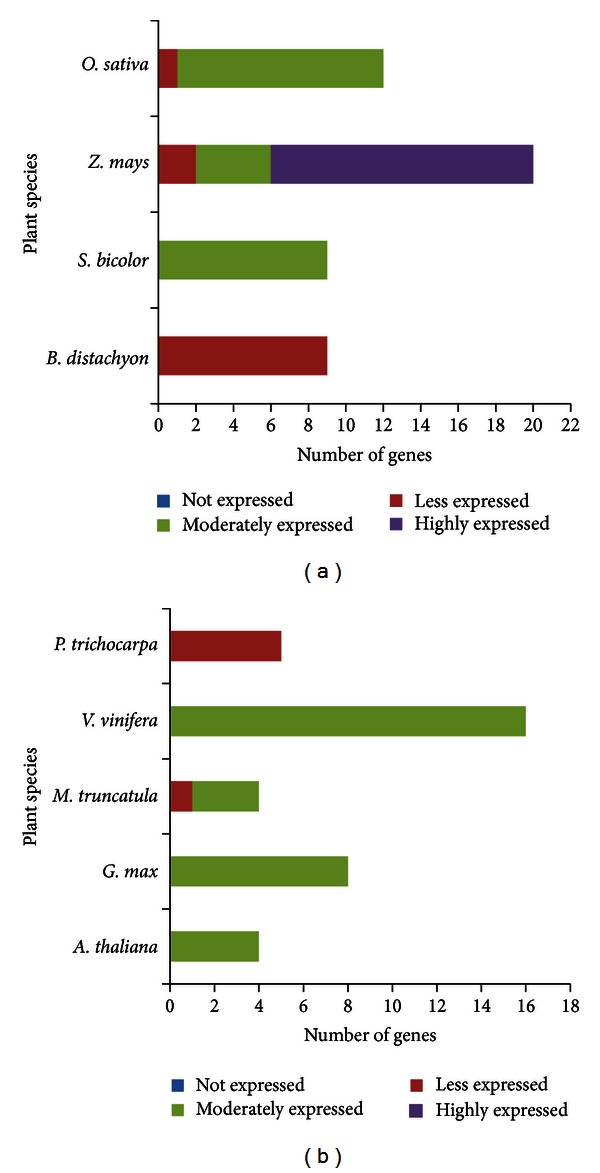
*In silico* expression analyses of PAL genes in (a) monocots and (b) dicots.

**Figure 3:**
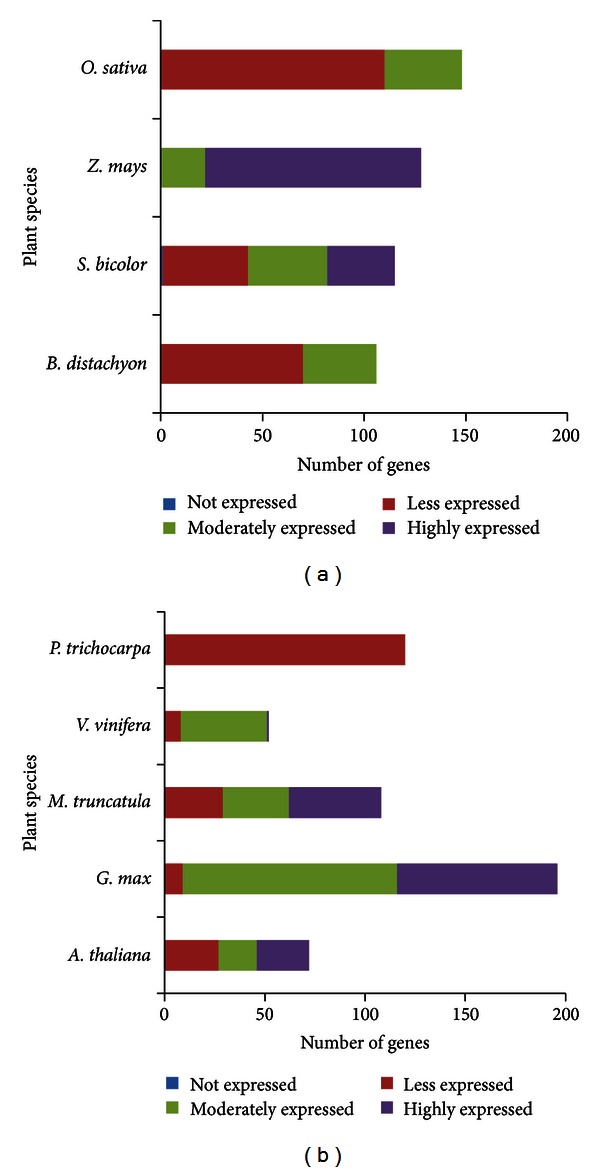
*In silico* expression analyses of POX A genes in (a) monocots and (b) dicots.

*In silico* expression analysis revealed that all POX A and PAL genes identified in the present study were found to be expressed, except one POX A gene in *Sorghum bicolor*. Based on the significant EST hits, we divided these expressed genes as less, moderately, or highly expressed genes. Among the identified 87 PAL genes, 79.31% were found to be either moderately or highly expressed, whereas 60.19% of 1045 POX A genes were observed to be moderately or highly expressed (Figures 2 and 3). 

### 3.2. Genome-Wide Distribution and Physical Mapping of *PAL* and *POX A* Genes

We performed BLAST search of the identified 87 PAL and 1045 POX A genes against the whole genome sequence of all seven plant genomes to find out their chromosomal position. Although PAL genes were restricted to few chromosomes, POX A genes were found to be distributed throughout the genomes and physically mapped on each chromosome of these species (see Figure S1 in Supplementary Material available online at http://dx.doi.org/10.1155/2013/678969). Many of these genes were present in major clusters. Interestingly, chromosome number 4 of both *Arabidopsis* and *Brachypodium* has the least number of these genes, but the same trend was observed in all nine plant species used in this study (see supplementary Figure S1). PAL genes were found absent or less in number on some chromosomes of monocots and *Medicago. *The* Medicago* has all PAL genes on chromosome 1, whereas none of other dicots were found to have PAL gene on chromosome 1. Hence, with respect to PAL genes, *Medicago* seems to follow the distribution pattern similar to that of monocots. In dicots, except *Medicago,* a higher percentage of PAL genes were found on some chromosomes, whereas on monocots, most of the PAL genes are present on chromosomes one to five. Many chromosomes of *Vitis* and *Populus* were not have PAL or POX A or both types of these genes.

Physical mapping indicated that the POX A genes are not only randomly distributed throughout the genomes but are also located in clusters and/or in subtelomeric regions of the chromosomes. The occurrence of clusters at subtelomere regions points toward the most significant feature of the proximal domain with the possibility of existence of POX A as the subtelomeric gene family and unusual high levels of sequence diversity among the member genes [19]. These clusters of POX A genes at telomere proximal regions may be linked to their rapid evolution [44]. Large number of identified genes and possible existence as subtelomeric gene family with clusters of genes at telomere proximal regions point toward the high evolution rate of POX A gene family as compared with that of the PAL gene family.

### 3.3. Phylogenetic Relation between *PAL* and *POX A* Genes of Different Species

**Figure 4:**
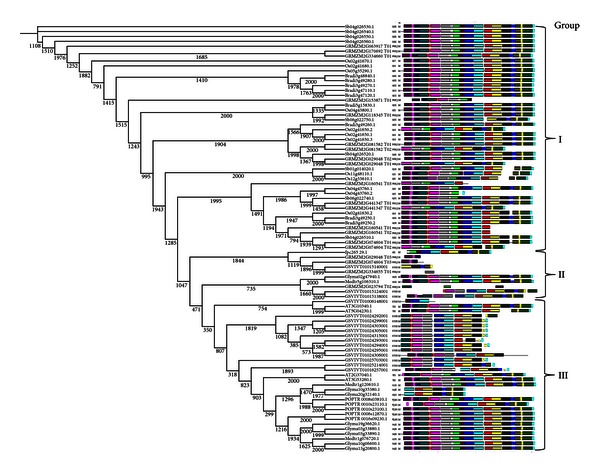
Schematic diagram of motif distribution of PAL genes. MEME 4.6.0 was applied to show that different subgroups were distinguished by the motif distribution, which is consistent with the phylogenetic subgroups obtained by ClustalX 2.1. Numbers written below each node are bootstrap values derived from 2000 replicates. Twenty conserved novel motifs were shown with different colored boxes.

The identified PAL and POX A genes not only represent two different DR categories but also belong to highly diverged plant species, ranging from monocots to dicots and including grains, grass, fruits, and legumes. We have analyzed these PAL and POX A genes to evaluate their evolutionary relations between monocots and dicots. For 87 PAL genes, MSA was obtained, and NJ distance tree was constructed and supported with bootstrap values using default settings and parameters. Motif distribution pattern was detected for these genes with MEME software, and an overlay was produced with the NJ tree as given in Figure 4.

A clear correlation between the motif pattern and the NJ tree can be found, where each group or subgroup of tree is essentially sharing the same motif pattern. Many motifs are more conserved and appeared in almost all groups or subgroups, except the ones at the middle portion of the tree. These conserved motifs could be the essential elements determining the PAL family's common molecular function among different plant species. Twelve of 20 maize PAL genes and all 16 *Vitis* PAL genes lack many motifs and might not be having the close evolutionary relations with other groups. The motif distribution revealed that the genes having the same motifs determined by MEME usually evolved from gene expansion within the same group or cluster whether they belong to higher or lower species [15]. It can be explained that the ancestor genes with various motif structure seem to appear early in the evolution, and then, the same structure was maintained by the recent genes through the evolution. In the present study, similar motif distribution points toward the conservation of the PAL genes throughout all of the groups and subgroups, except the two subgroups in the middle of the tree (Figure 4).

**Figure 5:**
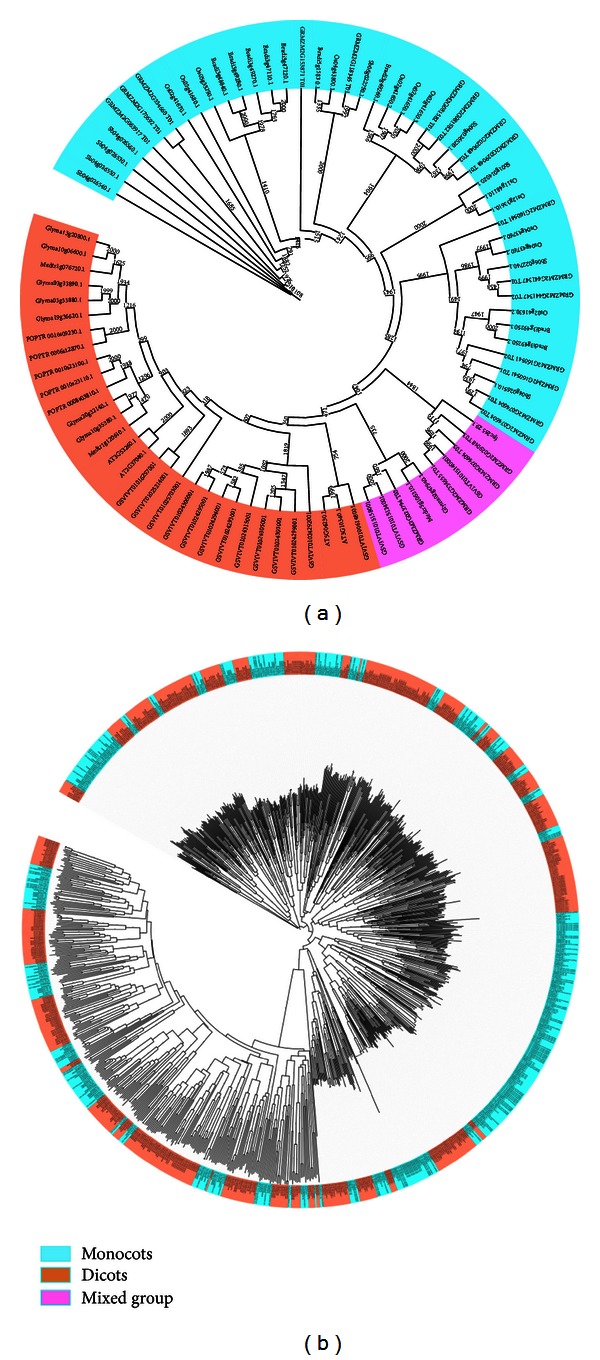
Phylogenetic tree of the (a) PAL family and the (b) POX A family. Amino acid sequences were aligned with ClustalW and NJ tree constructed using the ClustalX 2.1 and interactively designed with iTOL. Bootstrap values are assigned above the branches. Orange and sky blue indicate dicot and monocot clusters, respectively, whereas a pink one indicates a mixed cluster found only with PAL.

The tree was further represented interactively on iTOL and was found to form three big classes, assigned with three different colors and representing three clear groups—monocots (sky blue), dicots (orange), and a mixed group (pink) containing both dicots and monocots with significant bootstrap values (Figure 5(a)). Four PAL genes of maize and three of *Vitis* PAL genes, those lacking many motifs in MEME motif distribution, were found in a separate phylogenetic class or group along with two *Medicago *PAL genes (Figure 4). The well-distinguished and big clusters of monocots followed by dicots are well in conjugation with those formed by the motif distribution and point toward the conservation of PAL genes along with their evolution from monocots to dicots.

There were only four PAL genes in *Arabidopsis *genome, which were found to form two clusters (AT3G10340.1-AT5G04230.1 and AT2G37040.1-AT3G53260.1) as also reported earlier [45, 46].

A close review and analysis of the obtained large NJ tree for 1045 POX A genes indicate that there was not a single small subgroup having both dicots and monocots together (Figure 5(b)), like the one that appears for PAL genes. Even the smallest group/subgroup of monocots (sky blue) contains at least one gene from all of the four monocot species included in this study. Similar trend was observed for the groups representing POX A genes from dicots (orange). Hence, none of the single species can be considered as basal one or descendent one. It seems that during the expansion of the POX A gene family, many groups and subgroups might have evolved, resulting in a high level of functional divergence between the POX A gene copies in monocots and dicots.

### 3.4. Synteny Analysis and Orthology

**Figure 6:**
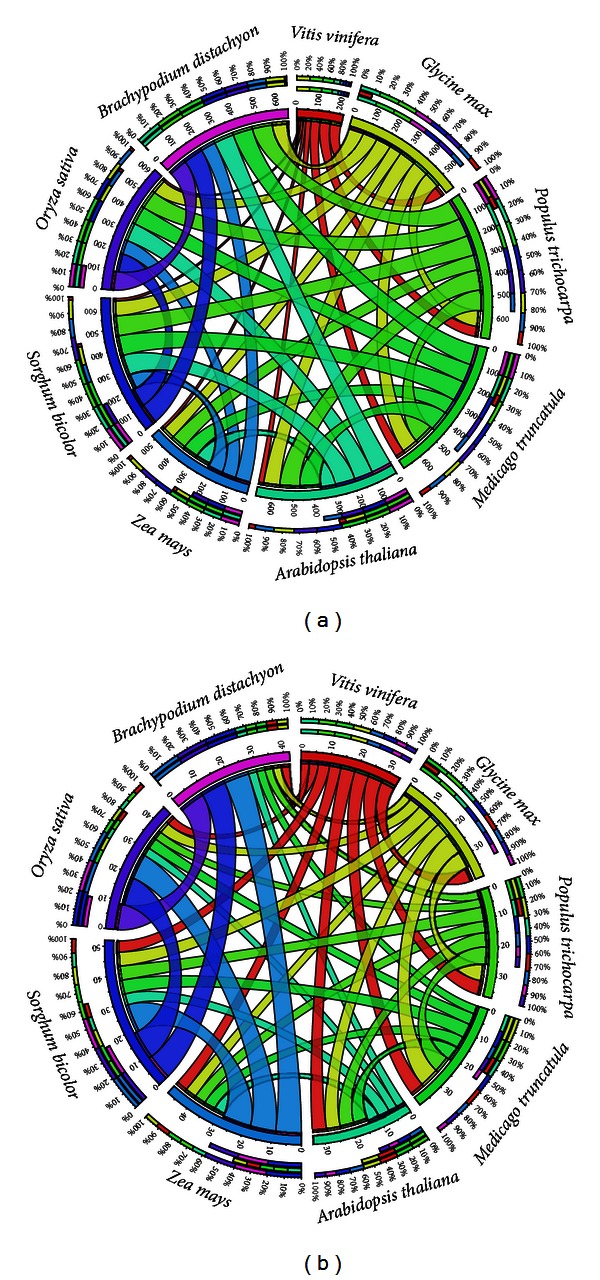
Orthologous pairs between different plant species for (a) PAL gene family and (b) POX A gene family. Each ribbon arising from a species (shown as clades) corresponds to the percentage of orthologous pairs with the destined species. PAL *Vitis *(red clade) was found to have the minimum orthology with other plant species. For POX A, monocots have high percentage of orthology, whereas *Vitis* and *Arabidopsis* were found to have the least orthology with others.

To infer the synteny, orthologous pairs were counted for PAL and POX A genes between each of the possible pair of species, as described in the methods. The orthology analysis revealed that for PAL genes, *Vitis vinifera *have the least orthology with other species (Figure 6(a)). Each ribbon arising from a species (shown as clades) corresponds to the percentage of orthologous pairs with the destined species. For instance, *Vitis *(red clade) was clearly found to have the minimum orthology with other plant species. Comparison of PAL genes identified in *Vitis* with the dicot genomes indicates that orthologous relations are well conserved: 25% with *Arabidopsis,* 51% with *Populu*s, 53% soybean, and 45% *Medicago*, whereas with monocots, the level of conservation of *Vitis* was very low: 13% with maize, 12% with* Sorghum*, 11% with rice, and 8% with *Brachypodium*. Maize PAL genes were found to have comparatively less orthology among monocots (73.3%) compared with other monocot species like *Sorghum* (89%), rice (84.66%), and *Brachypodium* (89%). Interestingly, PAL genes from *Medicago, *a dicot species, showed comparatively more orthological orientation toward monocots (91.75%) as compared with other dicots (81.75%). That is, again, it is *Medicago, *a dicot species, that seems to follow the orientation of monocot species and have an extra shift toward monocots as observed with gene distribution and phylogenetic analysis. Meanwhile a high percentage of orthology, except *Vitis, *points toward the high conservation of the PAL gene family among the plant species.

For POX A, although a very low percentage of orthologous pairs was found between all species, comparatively, it was slightly higher among monocots. Comparison of POX A genes indicates that orthologous relations are conserved for only 5 to 7% between dicots, whereas with monocots, it is only from 9 to 10% as can be seen with the wide ribbons (blue, purple, and violet) for monocot species (Figure 6(b)). A high number of gene were identified in POX A gene family with very low orthology with other species. It might be caused by a high rate of evolution and expansion, which brought a high level of functional divergence among the members.

## 4. Conclusions

With our comparative genomics, genomic distribution, and phylogenetic and synteny analyses between the various plant species, we proposed a model of evolution of the PAL and POX A gene family. Limited numbers of identified genes (i.e., 87) in the PAL family, similar motif patterns, well-distinguished monocot-dicot groups in the phylogenetic tree, and high percentage of orthology point toward two main conclusions: (i) this family predates monocot/eudicot divergence with hardly any expansion after monocot/eudicot divergence, and (ii) second is remarkable conservation of function. Although there is a uniform random distribution throughout the genomes with clusters at the subtelomeric regions, many alternate and specific phylogenetic group/subgroups for monocots and dicots and a very low percentage of orthology for POX A genes may suggest the following: (i) the possible existence of this family as a subtelomeric gene family (ii) possible expression of unusually high levels of sequence diversity (iii) higher rate of evolvement and expansion than the PAL gene family and (iv) evolution of many groups and subgroups during the expansion of the family, resulting in a high level of functional divergence.

## Supplementary Material

Figure S1: Physical mapping of PAL and POXA genes in Arabidopsis thaliana; Brachypodium distachyon; Glycine max; Medicago truncatula; Vitis vinifera; Populus trichocarpa; Zea mays; Oryza sativa and Sorghum bicolor. Physical positions of genes are given in base pairs on left side. Gene ID and their forward (*►*) and reverse (*◄*) directions are shown on right side. Chromosome length is given on the top. Colour codes indicate PAL (green) and POXA (pink) genes.

## Figures and Tables

**Table 1:** List of plant species, chromosome number, genome size, and number of genes and ESTs.

S. no.	Crop	Chromosomes	Genome size*	Gene*	ESTs*
1	*Arabidopsis thaliana *	5	119.14 MB	33,410	15,29,262
2	*Glycine max *	20	950.06 MB	46,430	14,59,820
3	*Medicago truncatula *	9	278.68 MB	53,425	2,69,238
4	*Vitis vinifera *	19	303.08 MB	26,346	3,62,193
5	*Populus trichocarpa *	19	307.84 MB	45,778	89,943
6	*Brachypodium distachyon *	5	271.14 MB	32,255	1,28,092
7	*Sorghum bicolor *	10	659.22 MB	36,338	2,09,828
8	*Zea mays *	10	2.06 GB	53,764	20,19,105
9	*Oryza sativa *	12	372.31 MB	67,393	2,02,458

*Data resource: PlantGDB (ftp://ftp.plantgdb.org/download/Genomes/); Phytozome (http://www.phytozome.net/).
