# Supplementary material for: Conservation, Divergence, and Genome-Wide Distribution of PAL and POX A Gene Families in Plants
Source: Int J Genomics. 2013 Mar 10;2013:678969. doi: 10.1155/2013/678969 (PMC3647544; doi:10.1155/2013/678969)
Supplement: Supplementary file 1 — Figure S1: Physical mapping of PAL and POXA genes in Arabidopsis thaliana; Brachypodium distachyon; Glycine max; Medicago truncatula; Vitis vinifera; Populus trichocarpa; Zea mays; Oryza sativa and Sorghum bicolor. Physical positions of genes are given in base pairs on left side. Gene ID and their forward (►) and reverse (◄) directions are shown on right side. Chromosome length is given on the top. Colour codes indicate PAL (green) and POXA (pink) genes. [file 678969.f1.pdf]

Physical mapping of PAL and POX A genes in *Arabidopsis thaliana; Brachypodium distachyon*; *Glycine max; Medicago truncatula; Vitis vinifera; Populus trichocarpa; Zea mays; Oryza sativa and Sorghum bicolor.* Physical positions of genes are given in base pairs on left side. Gene ID and their forward and reverse directions are shown on right side. Chromosome length is given on the top. Colour codes indicates PAL (green) and POX A (pink) genes**.**

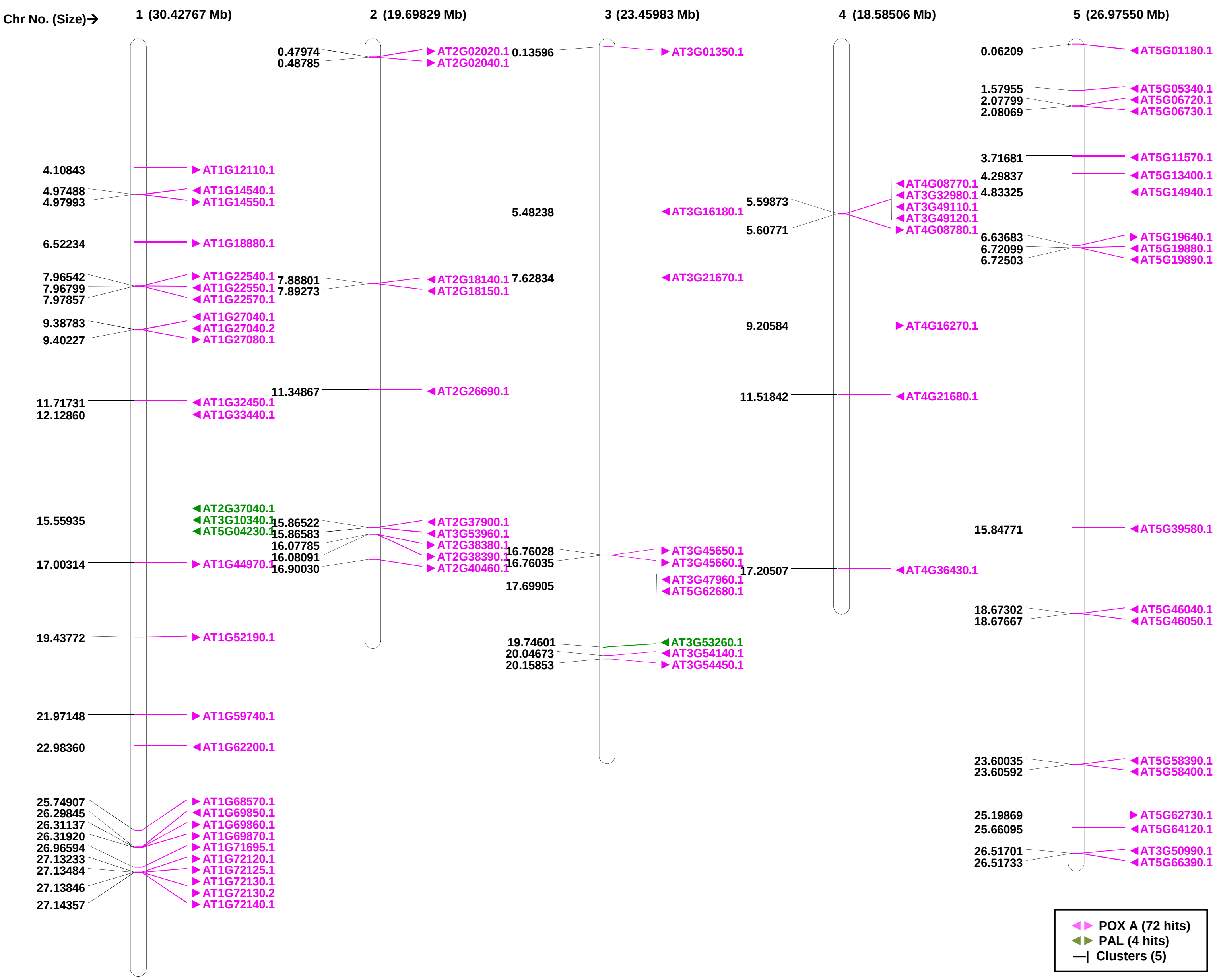

Physical mapping of PAL and POX A genes in *Arabidopsis thaliana*

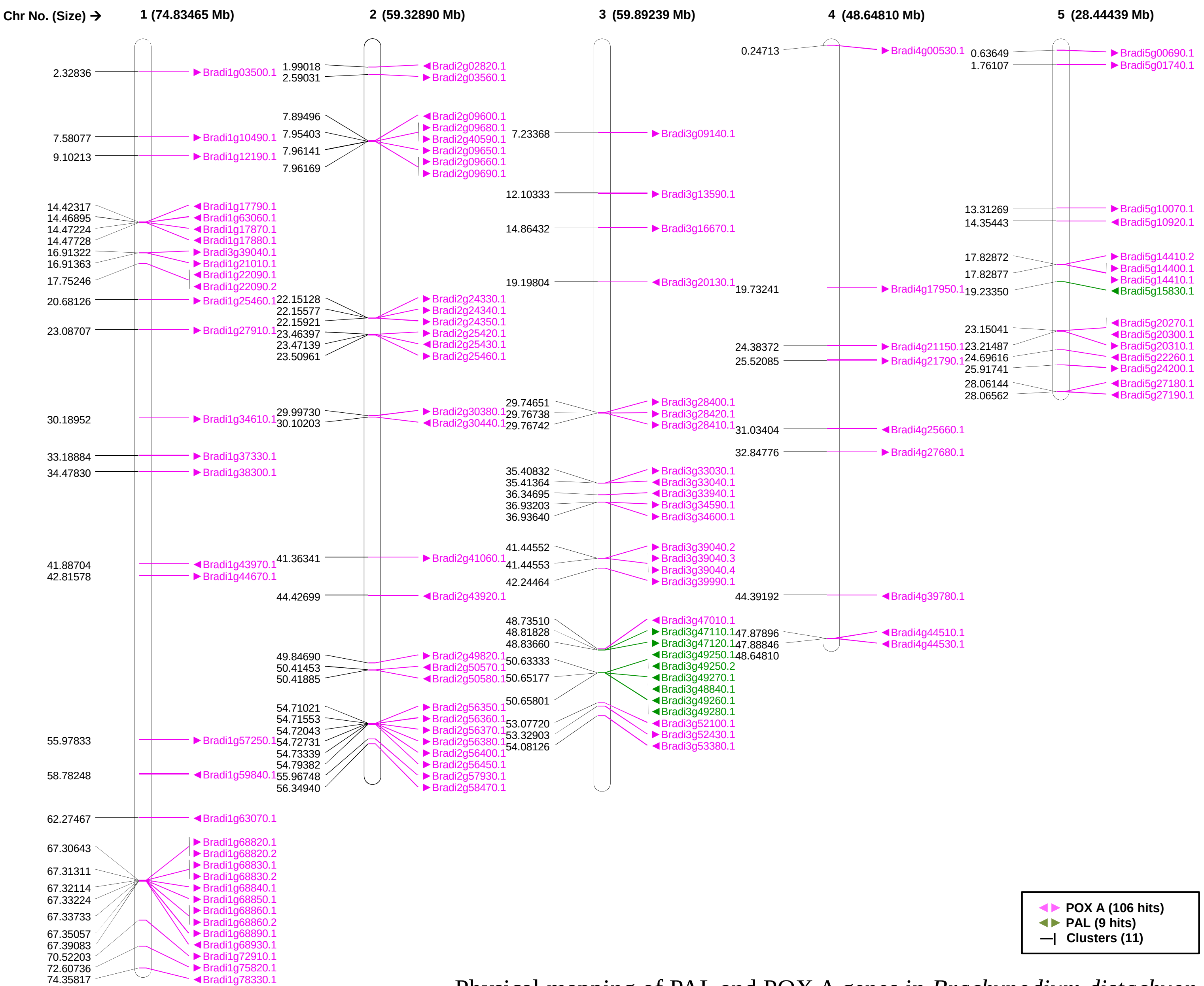

Physical mapping of PAL and POX A genes in *Brachypodium distachyon*

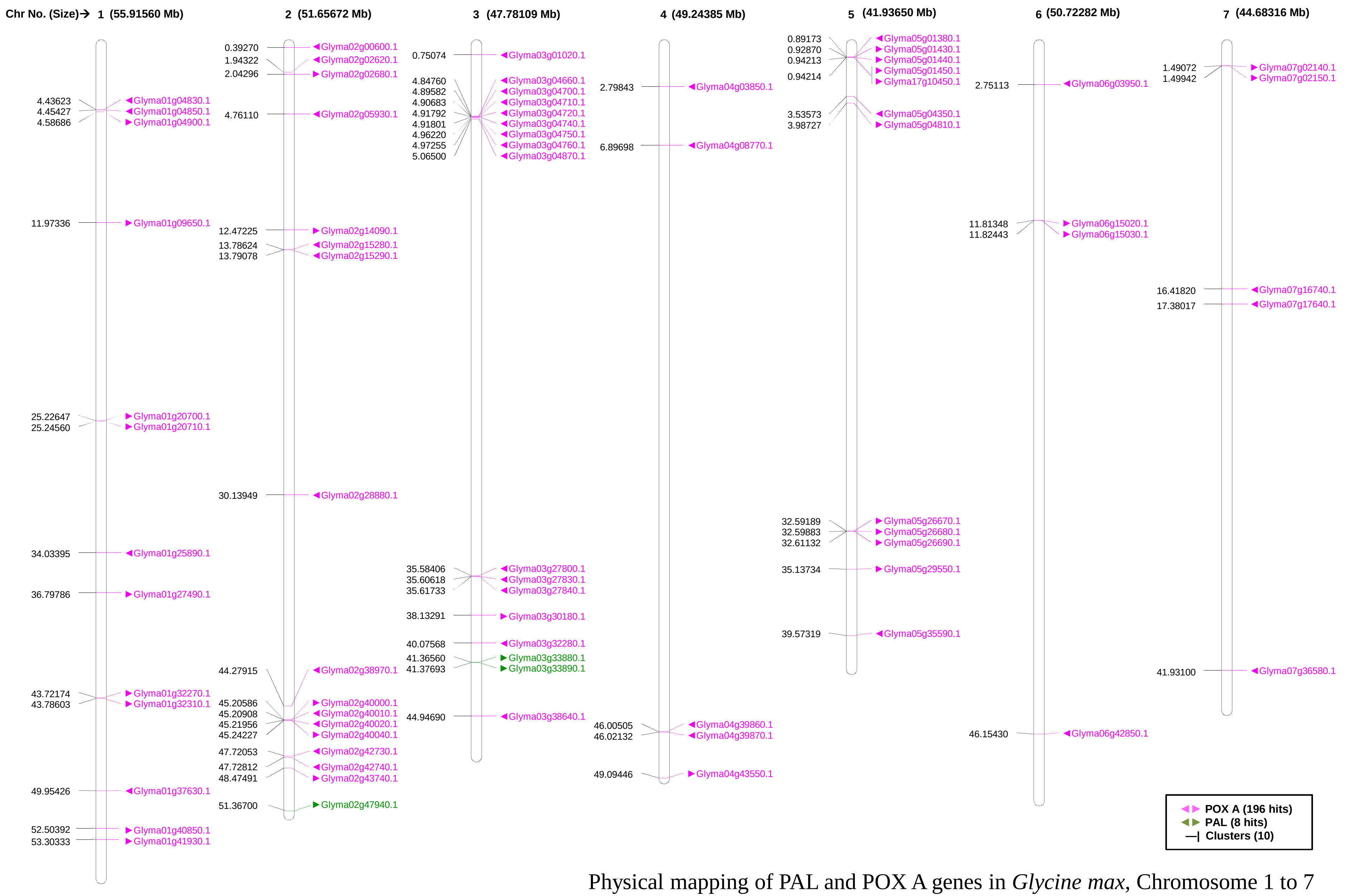

Physical mapping of PAL and POX A genes in *Glycine max*, Chromosome 1 to 7

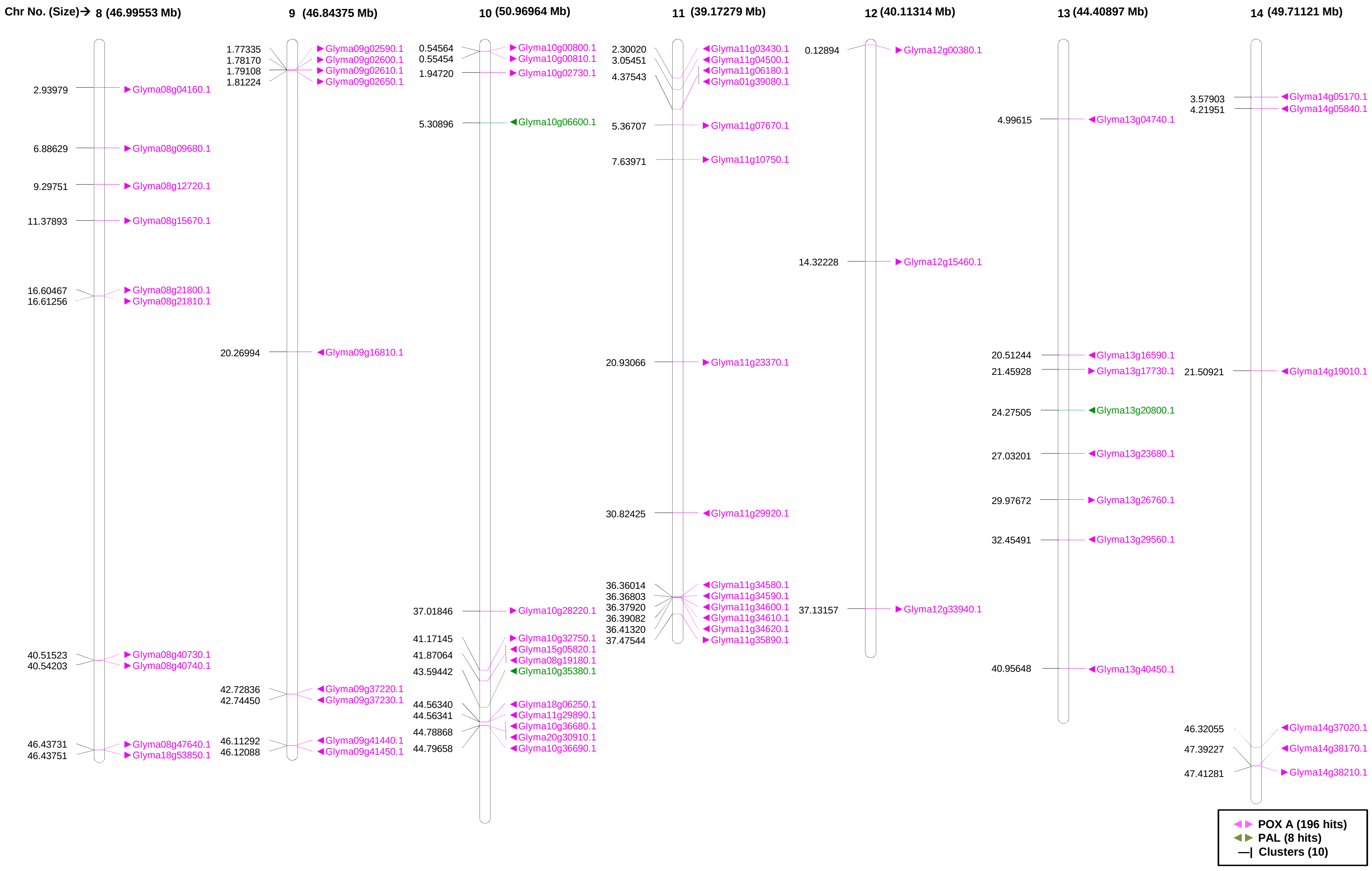

Physical mapping of PAL and POX A genes in *Glycine max*, Chromosome 7 to 14

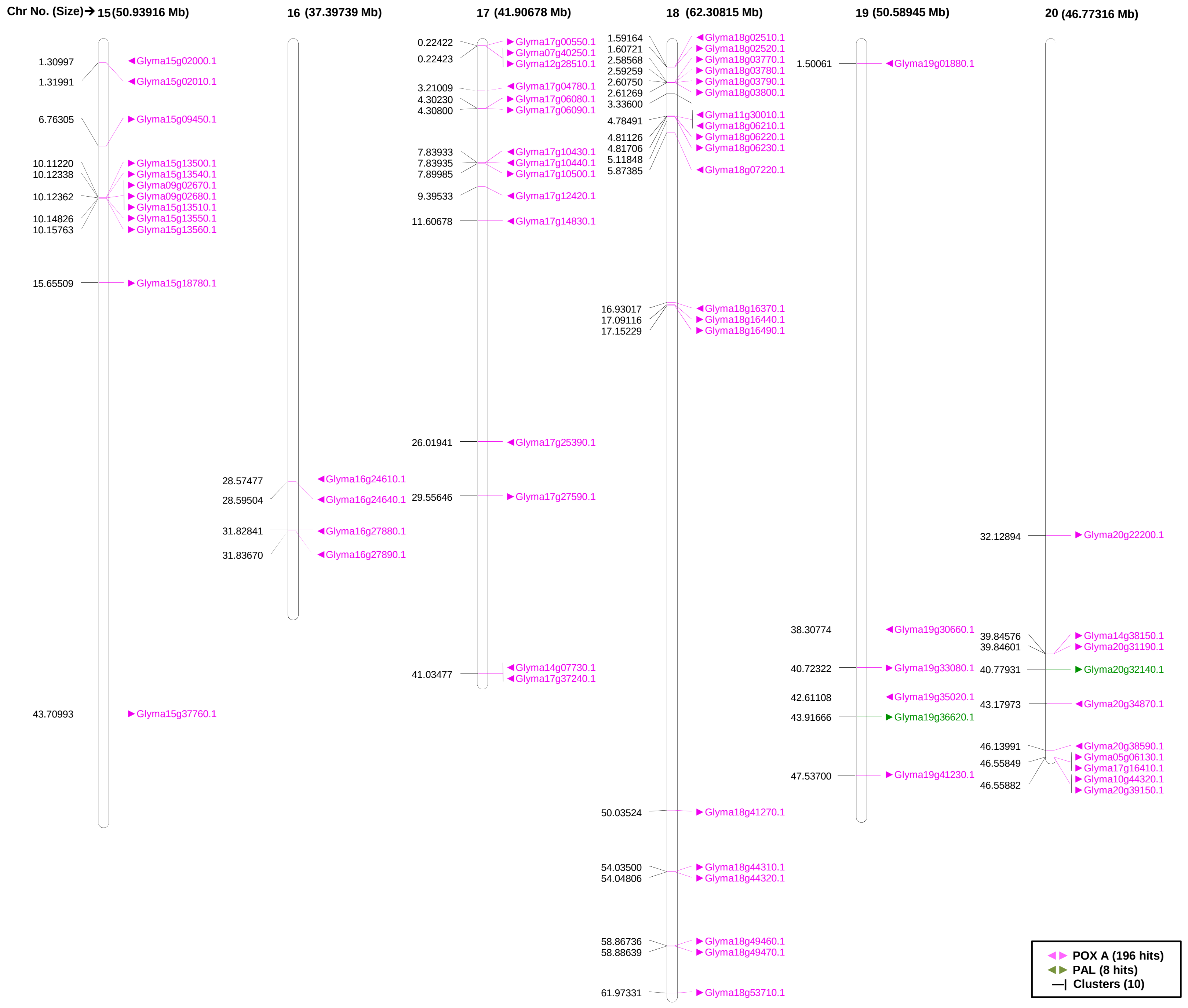

Physical mapping of PAL and POX A genes in *Glycine max* Chromosome 15 to 20

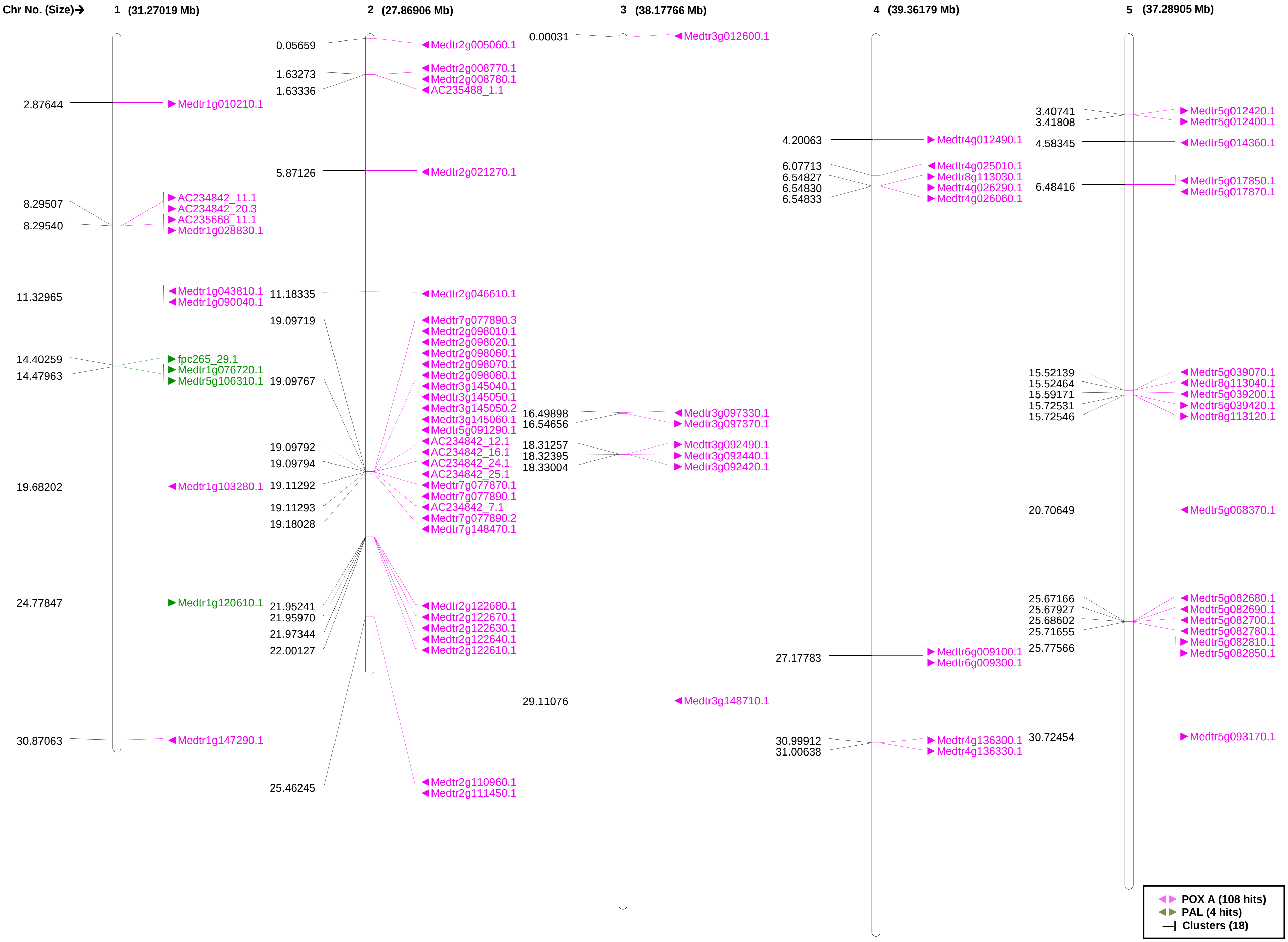

Physical mapping of PAL and POX A genes in *Medicago truncatula*, Chromosome 1 to 5

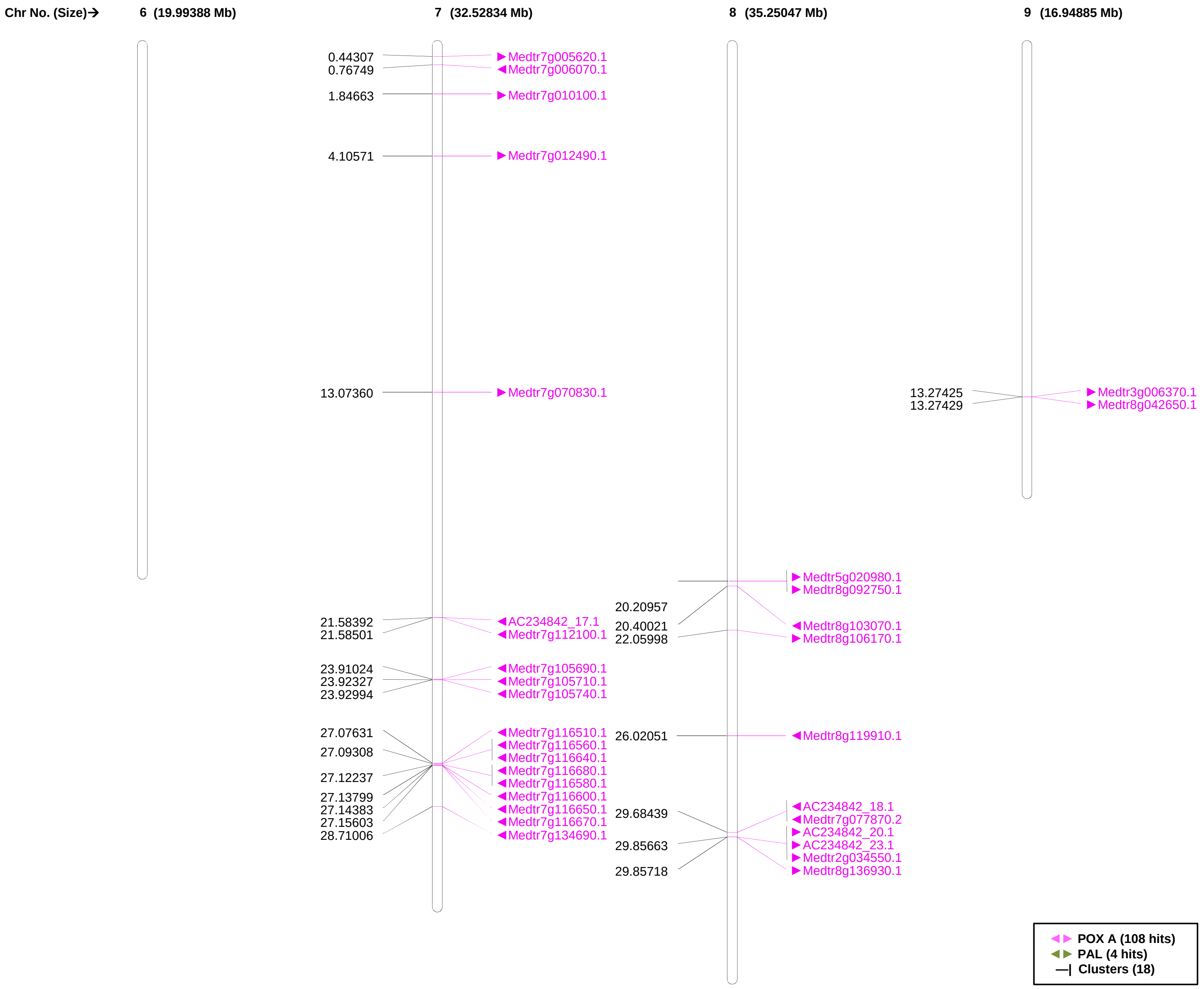

Physical mapping of PAL and POX A genes in *Medicago truncatula*, Chromosome 6 to 9

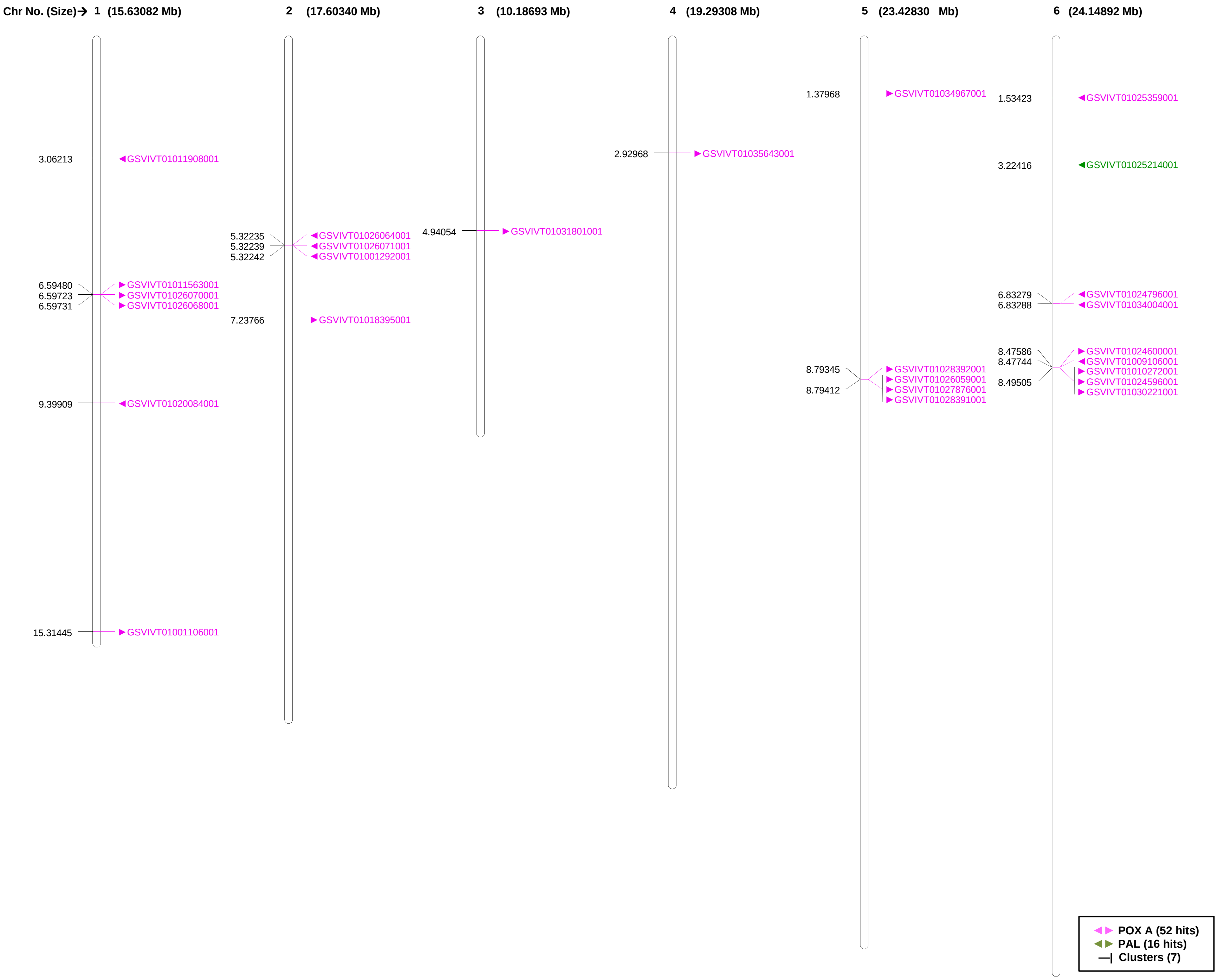

Physical mapping of PAL and POX A genes in *Vitis vinifera*, Chromosome 1 to 6

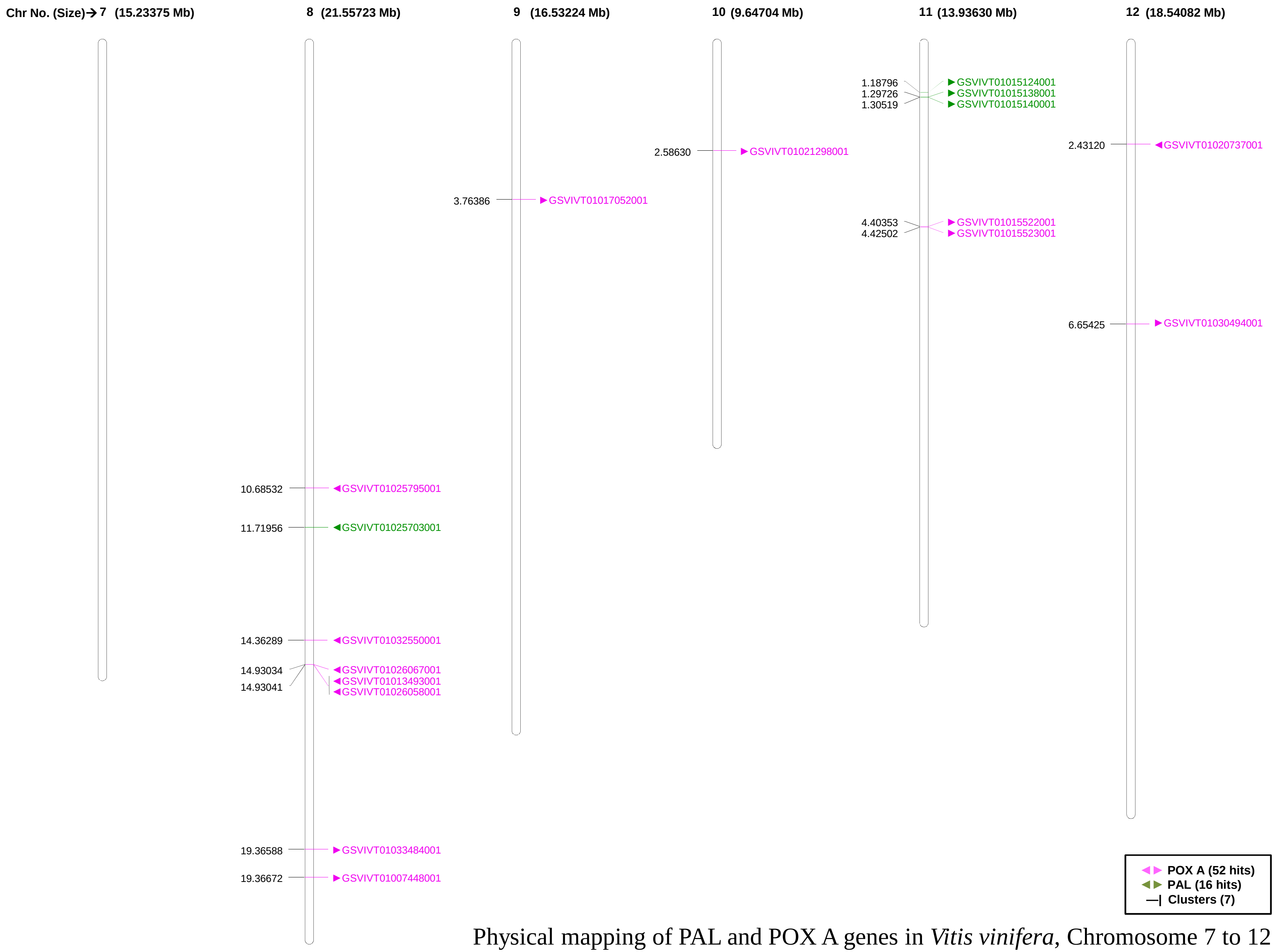

Physical mapping of PAL and POX A genes in *Vitis vinifera*, Chromosome 7 to 12

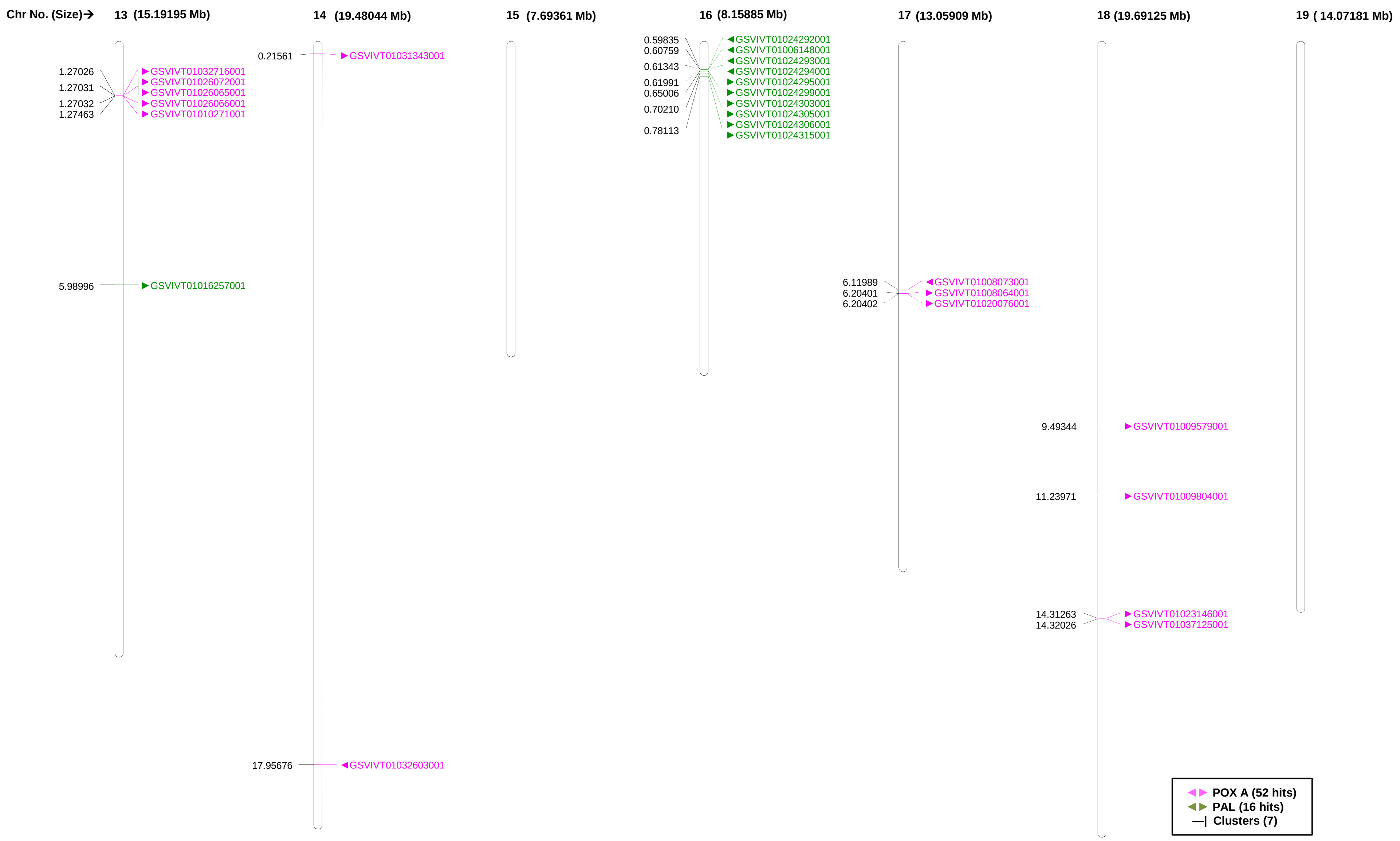

Physical mapping of PAL and POX A genes in *Vitis vinifera*, Chromosome 13 to 19

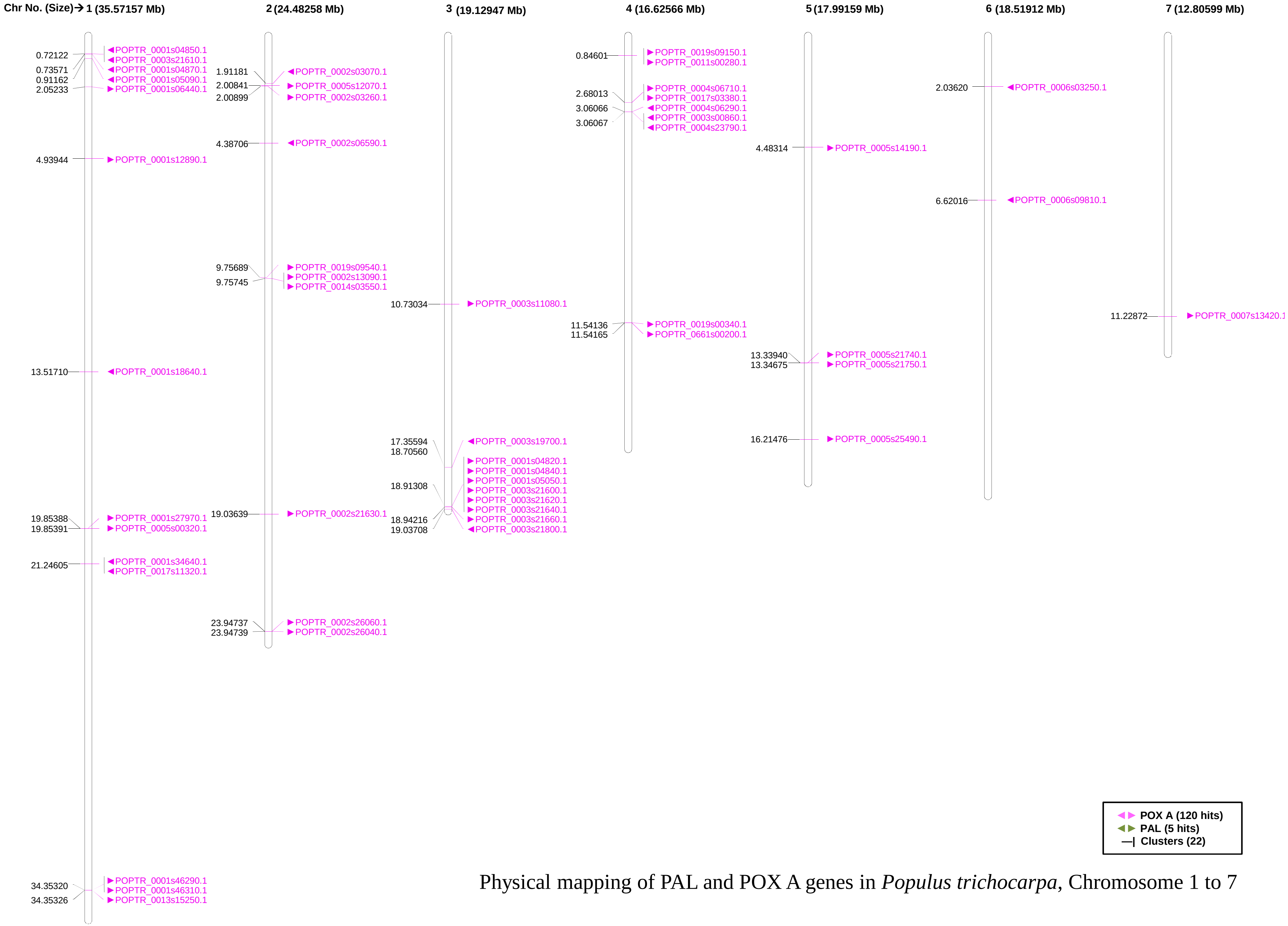

Physical mapping of PAL and POX A genes in *Populus trichocarpa*, Chromosome 1 to 7

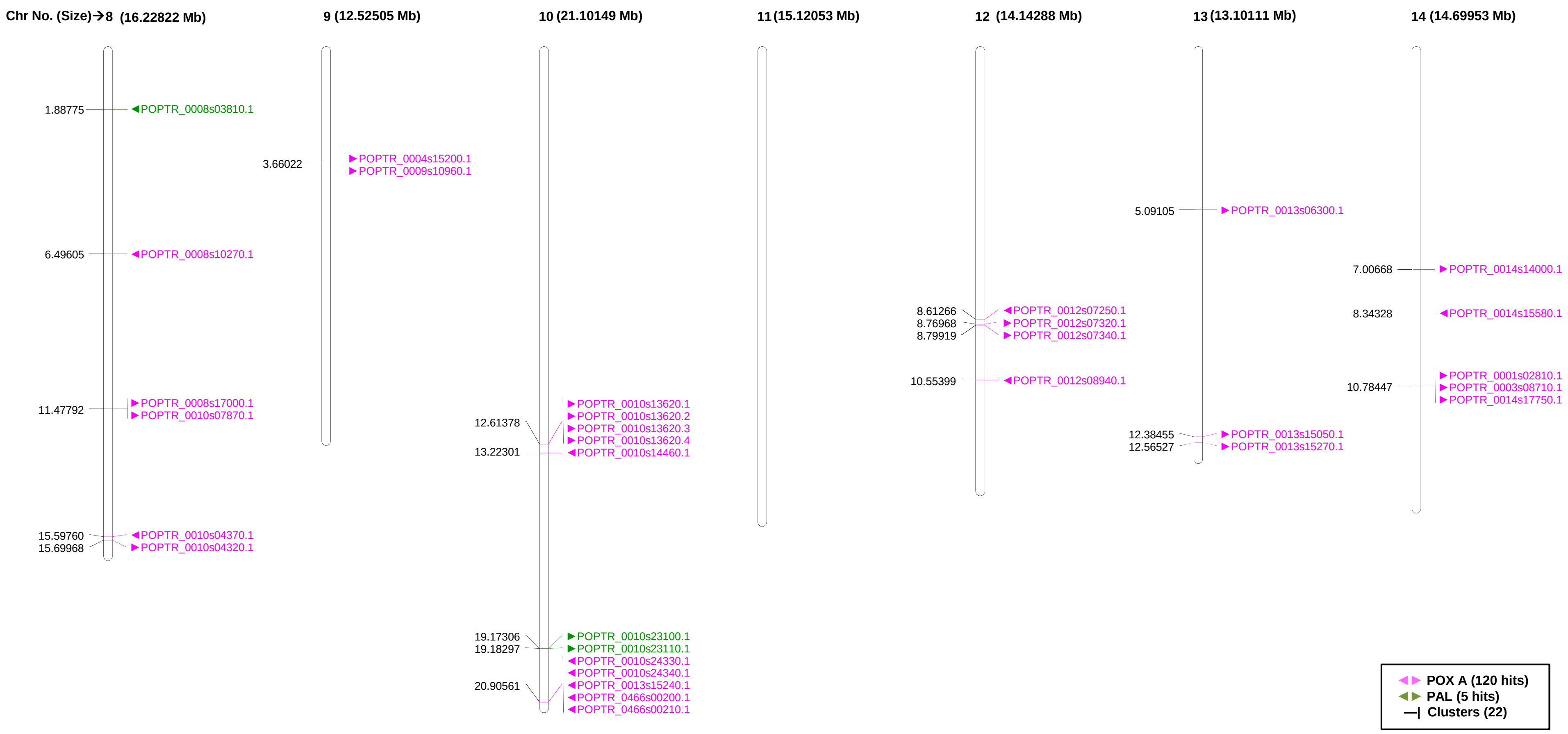

Physical mapping of PAL and POX A genes in *Populus trichocarpa*, Chromosome 8 to 14

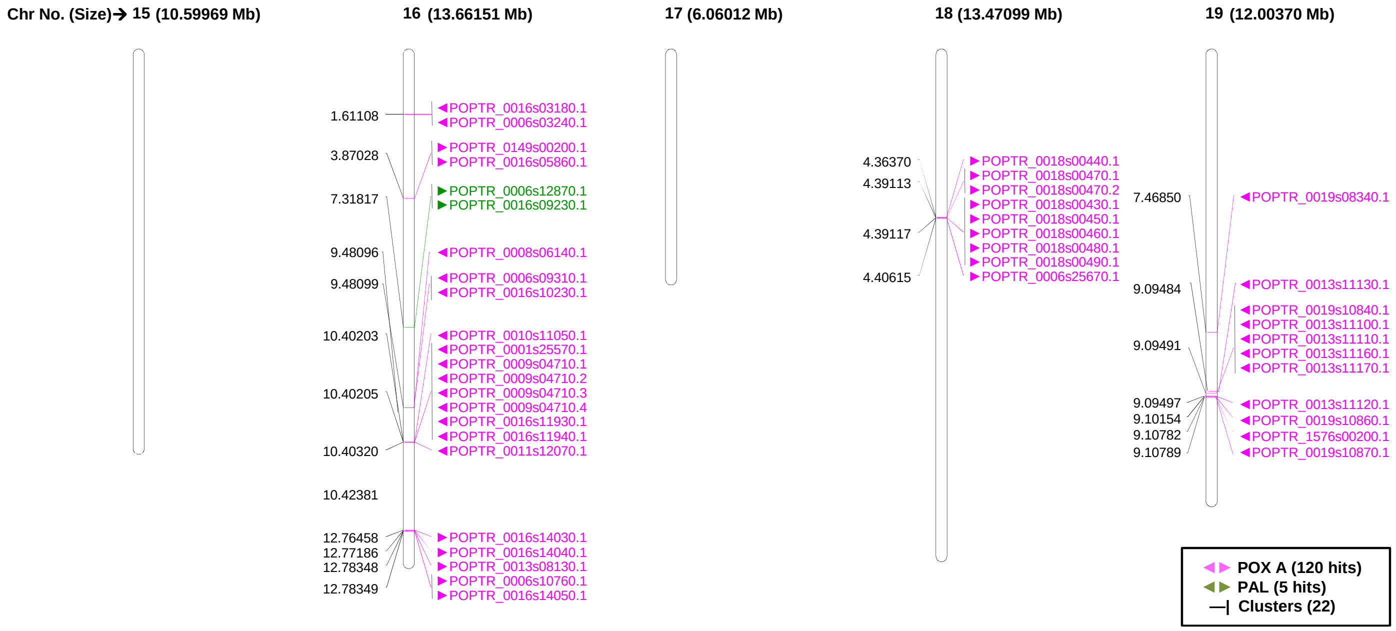

Physical mapping of PAL and POX A genes in *Populus trichocarpa*, Chromosome 15 to 19

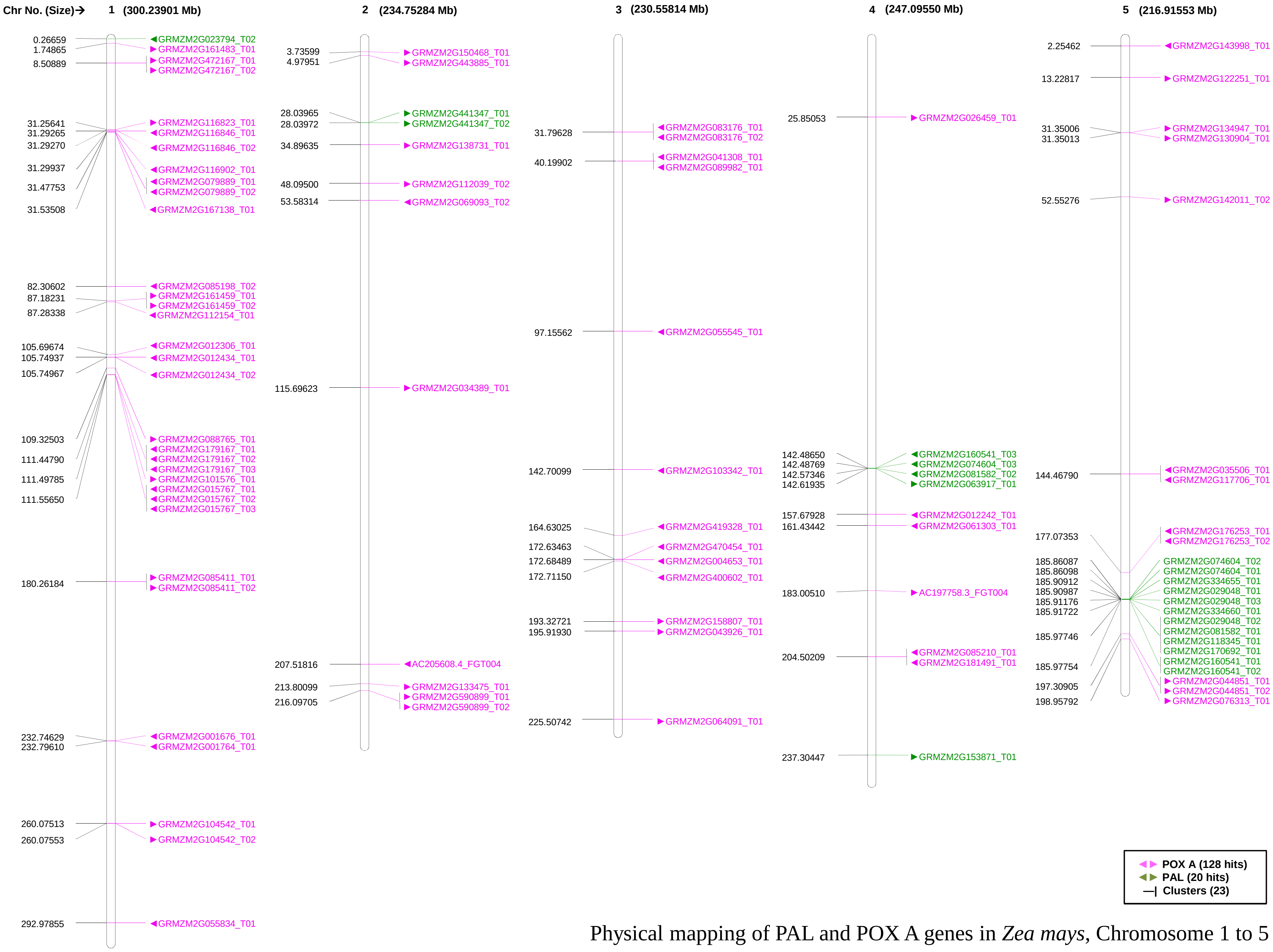

Physical mapping of PAL and POX A genes in *Zea mays*, Chromosome 1 to 5

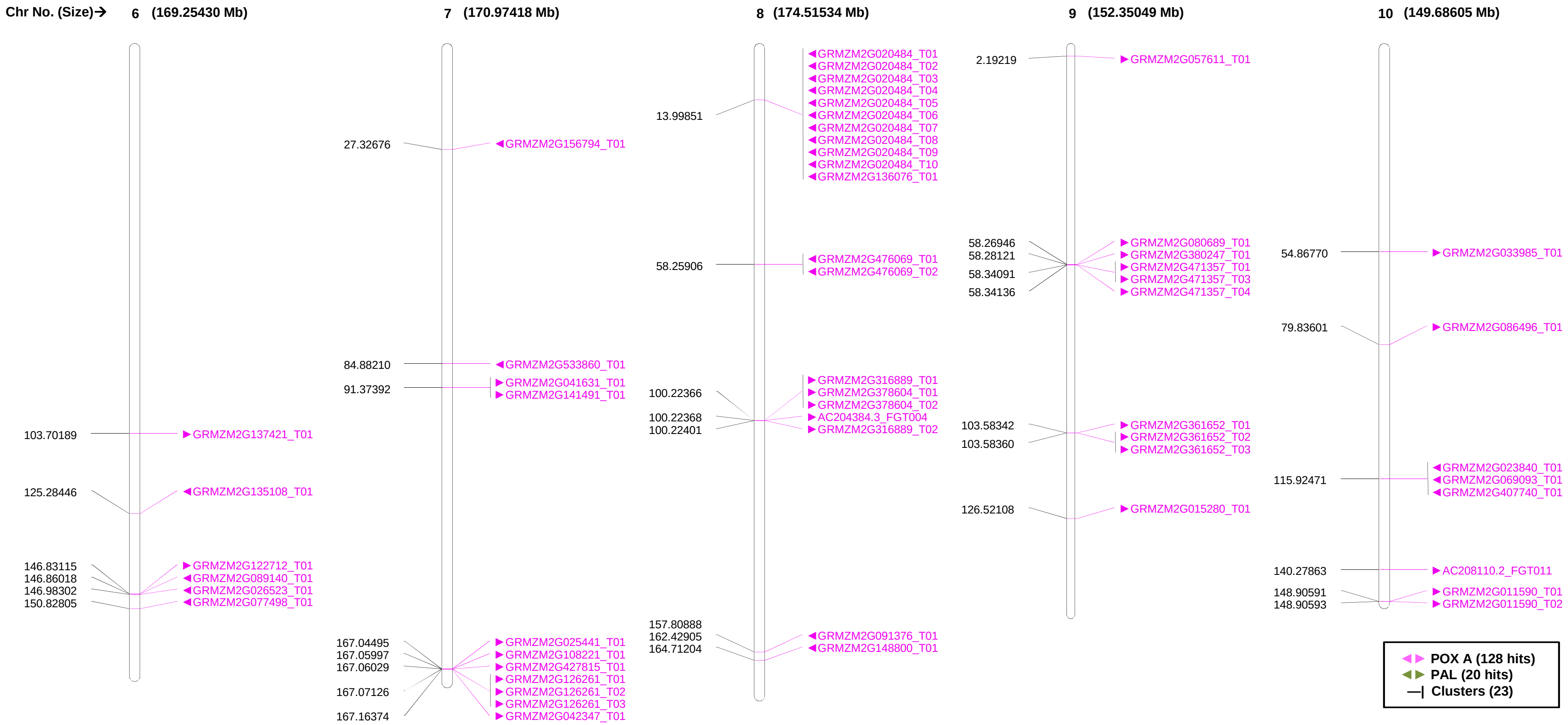

Physical mapping of PAL and POX A genes in *Zea mays*, Chromosome 5 to 10

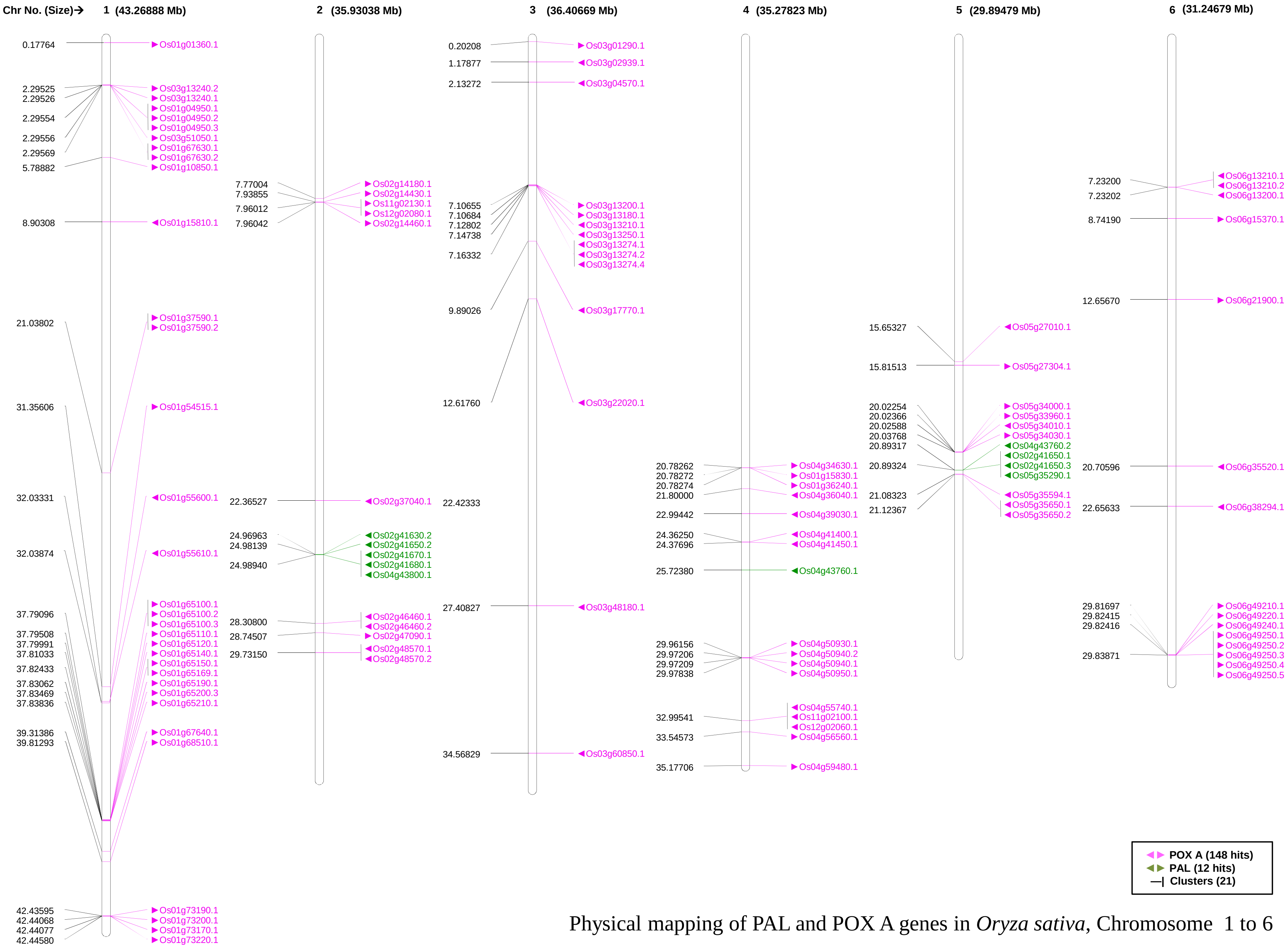

Physical mapping of PAL and POX A genes in *Oryza sativa*, Chromosome 1 to 6

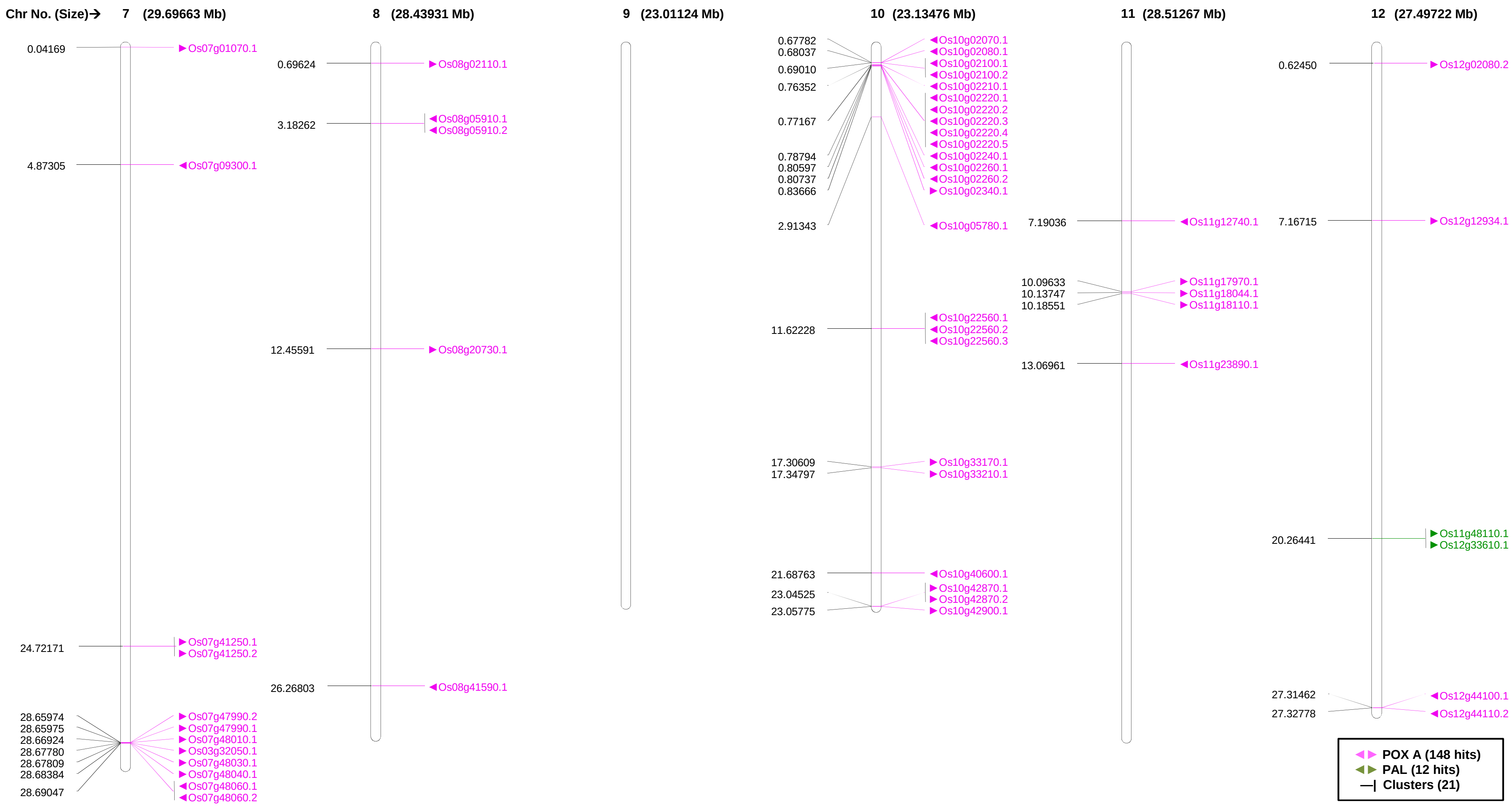

Physical mapping of PAL and POX A genes in *Oryza sativa*, Chromosome 7 to 12

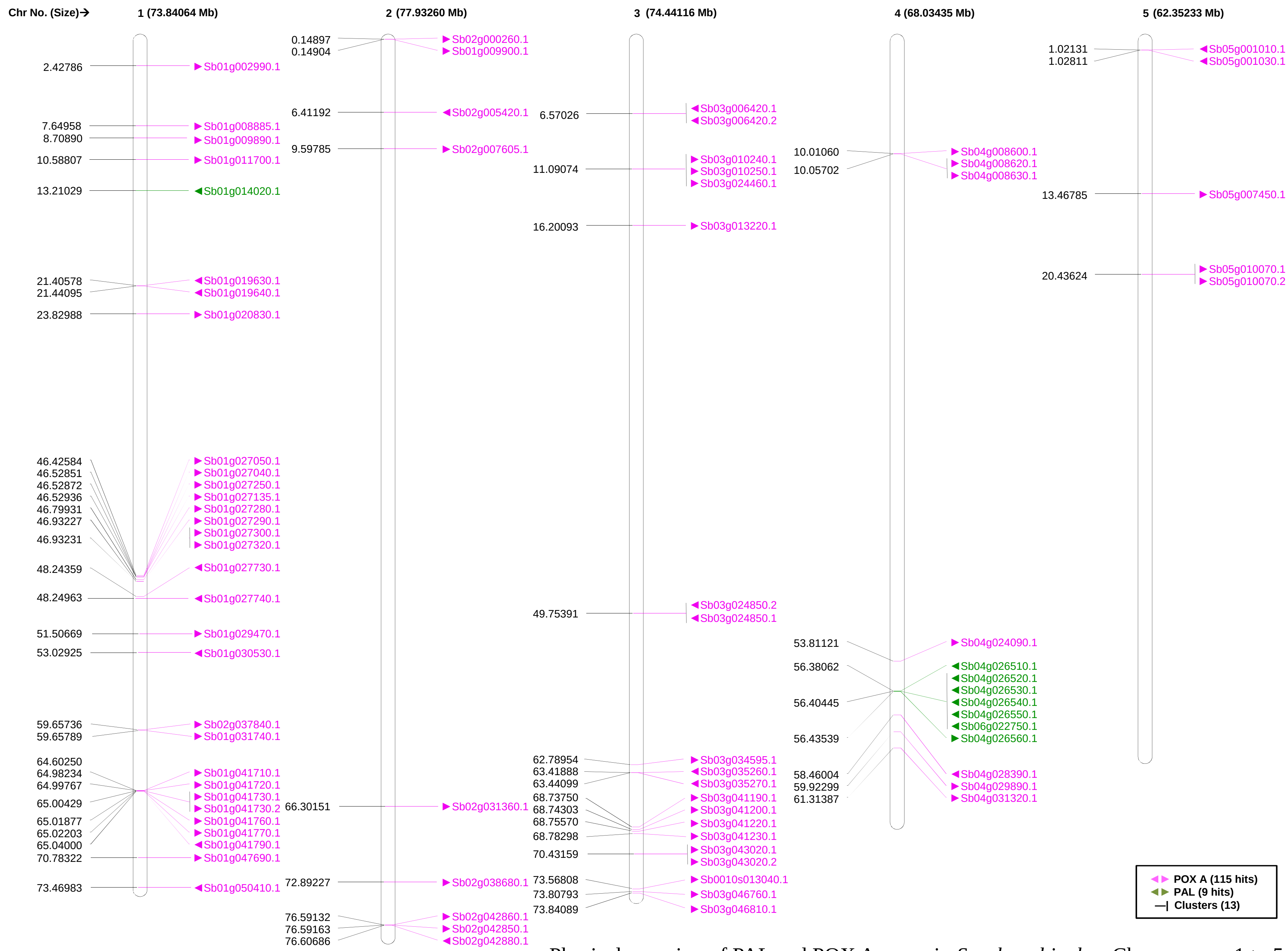

Physical mapping of PAL and POX A genes in *Sorghum bicolor*, Chromosome 1 to 5

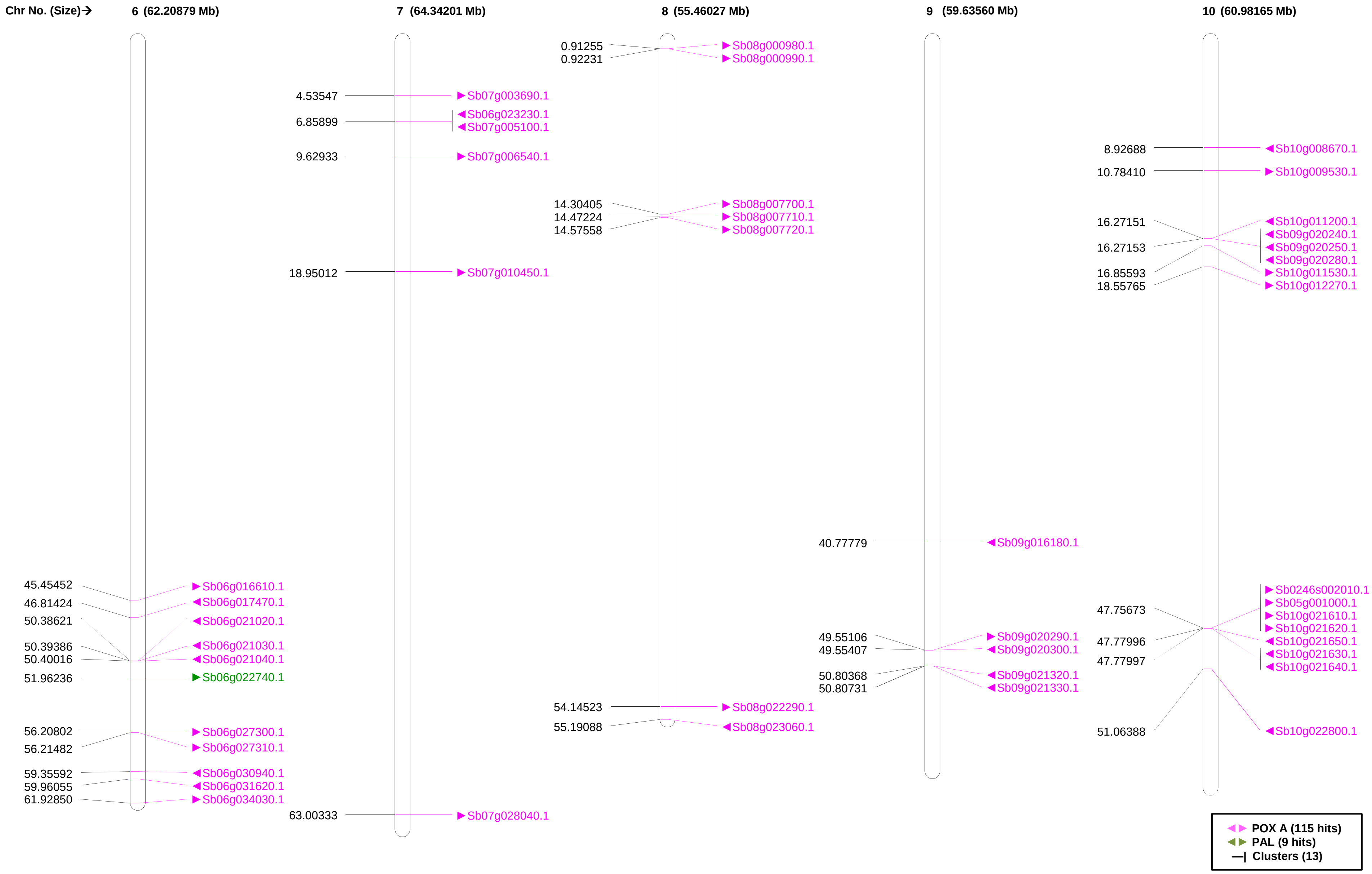

Physical mapping of PAL and POX A genes in *Sorghum bicolor*, Chromosome 6 to 10
